# Supplementary material for: Vitamin interdependencies predicted by metagenomics-informed network analyses and validated in microbial community microcosms
Source: Nat Commun. 2023 Aug 8;14:4768. doi: 10.1038/s41467-023-40360-4 (PMC10409787; doi:10.1038/s41467-023-40360-4)
Supplement: Supplementary file 1 — Supplementary Information [file 41467_2023_40360_MOESM1_ESM.pdf]

Supplementary information

Vitamin interdependencies predicted by metagenomics-informed network analyses and validated in microbial community microcosms

Supplementary figures

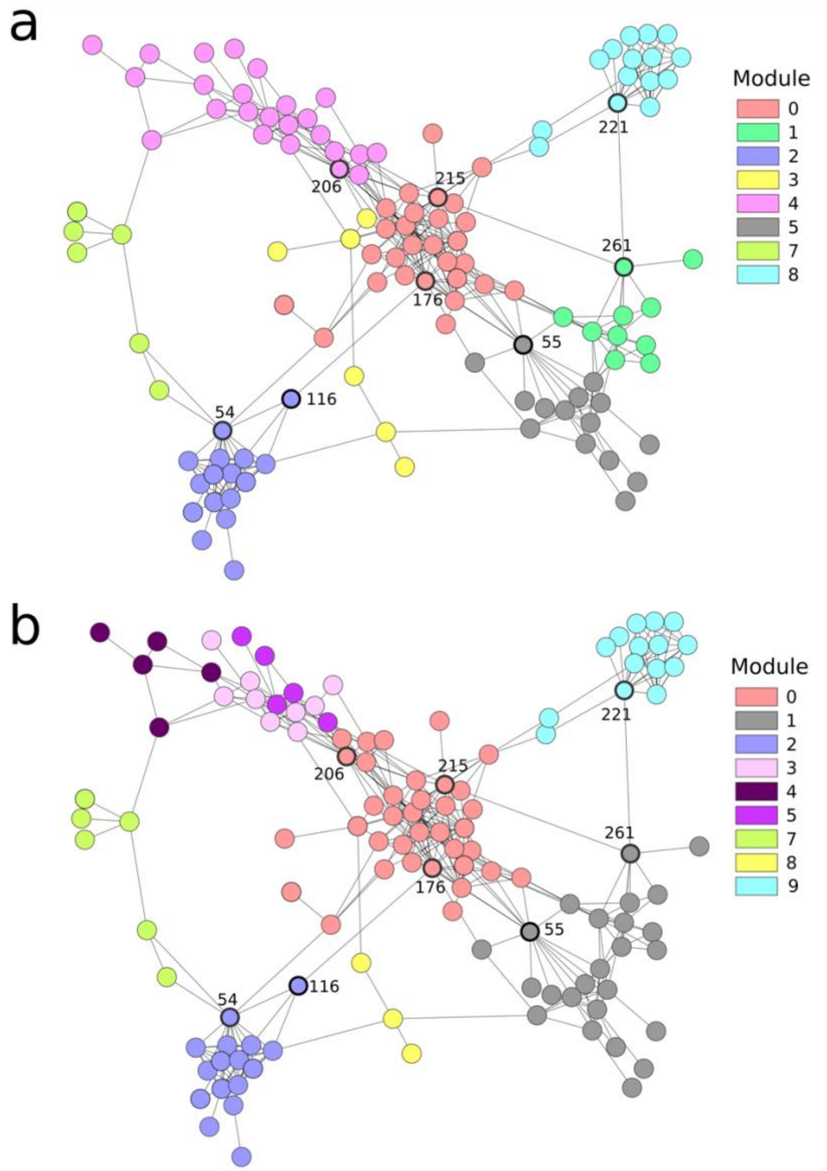

Figure S1. The determined modules of the correlation network, as shown in Figure 2A, produced using the (A) Louvian and (B) Girvan-Newman algorythmns. Network modules are shown in differing colors and the delineated network hub are shown with bold node borders and node numbers. The modules produced using the Louvian algorithm are refered to in the Results and the Discussion sections. See Tabls S2 for further network and node information.

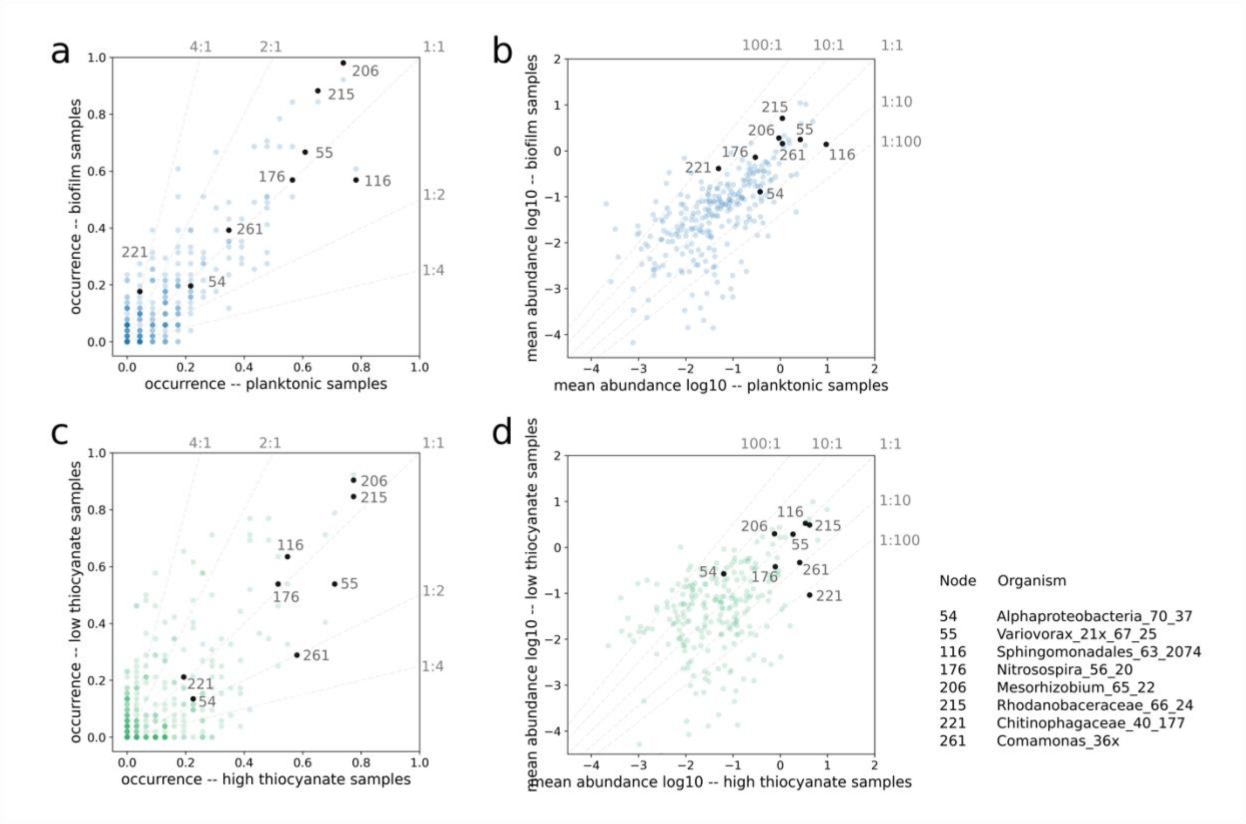

Figure S2. The mean abundance (a,c) and the frequency (b,d) of microorganism occurring in the planktonic versus biofilm communities (a,b) and the communities subjected to low (=< 250 mg/L) versus high (>250 mg/L) thiocyanate concentrations (c,d). n=92 biologically independent The nodes delineated as network hubs are shown in black and annotated with their node numbers. Occurrence is defined as the frequency of an organism occurring within a community at or greater to 0.1% relative abundance divided by the total number of samples under that condition. N=23 for planktonic, n=51 for biofilm biologically independent samples, and n=31 for high thiocyanate and n=52 for low thiocyanate biologically independent samples.

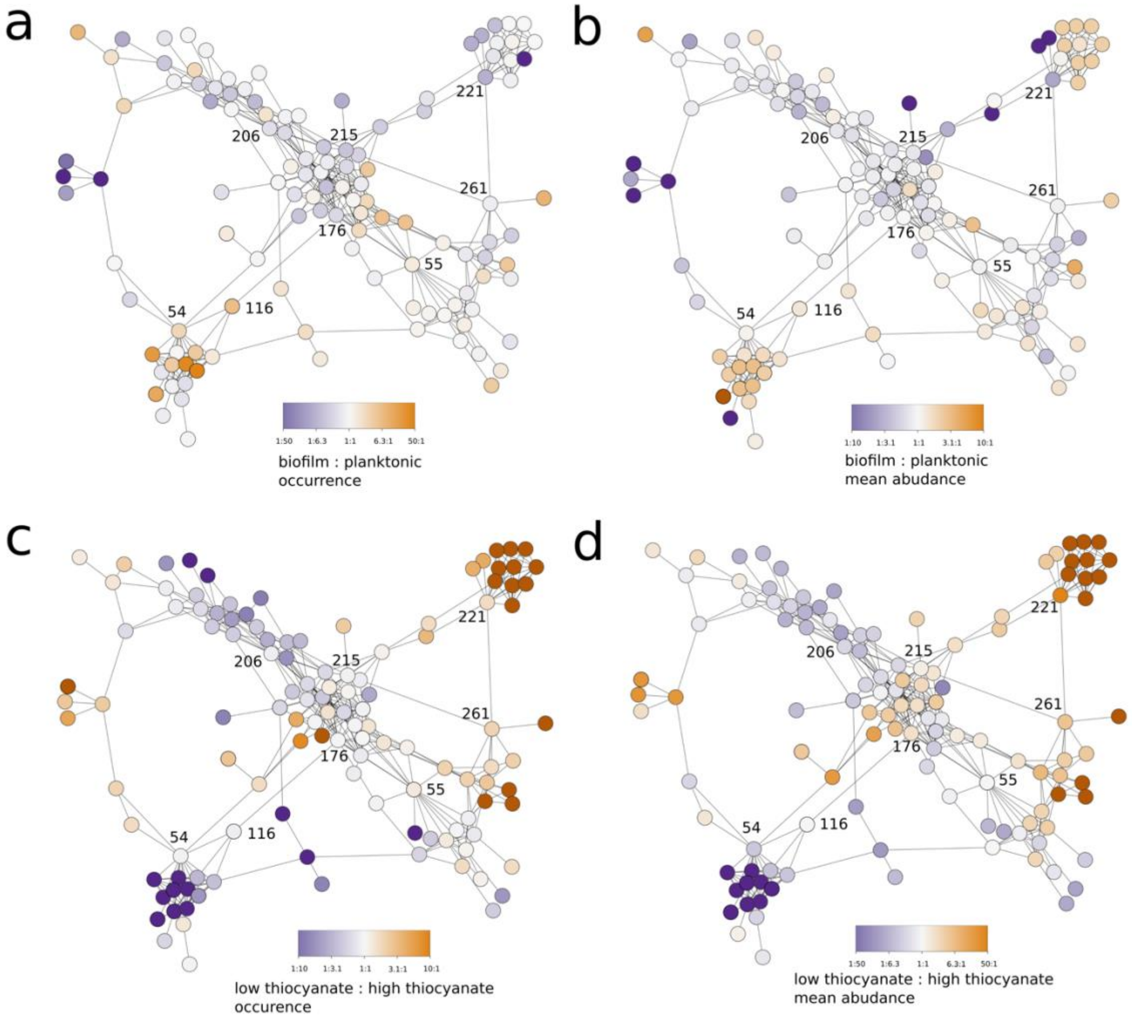

Figure S3. The Thiocyanate bioreactor communities correlation networks overlaid with information relating to the mean abundance (a,c) and the frequency (b,d) of microorganism occurring in the planktonic versus biofilm communities (a,b) and the communities subjected to low (=< 250 mg/L) versus high (>250 mg/L) thiocyanate concentrations (c,d), as shown in Figure S2. The nodes delineated as network hubs are annotated with their node numbers.

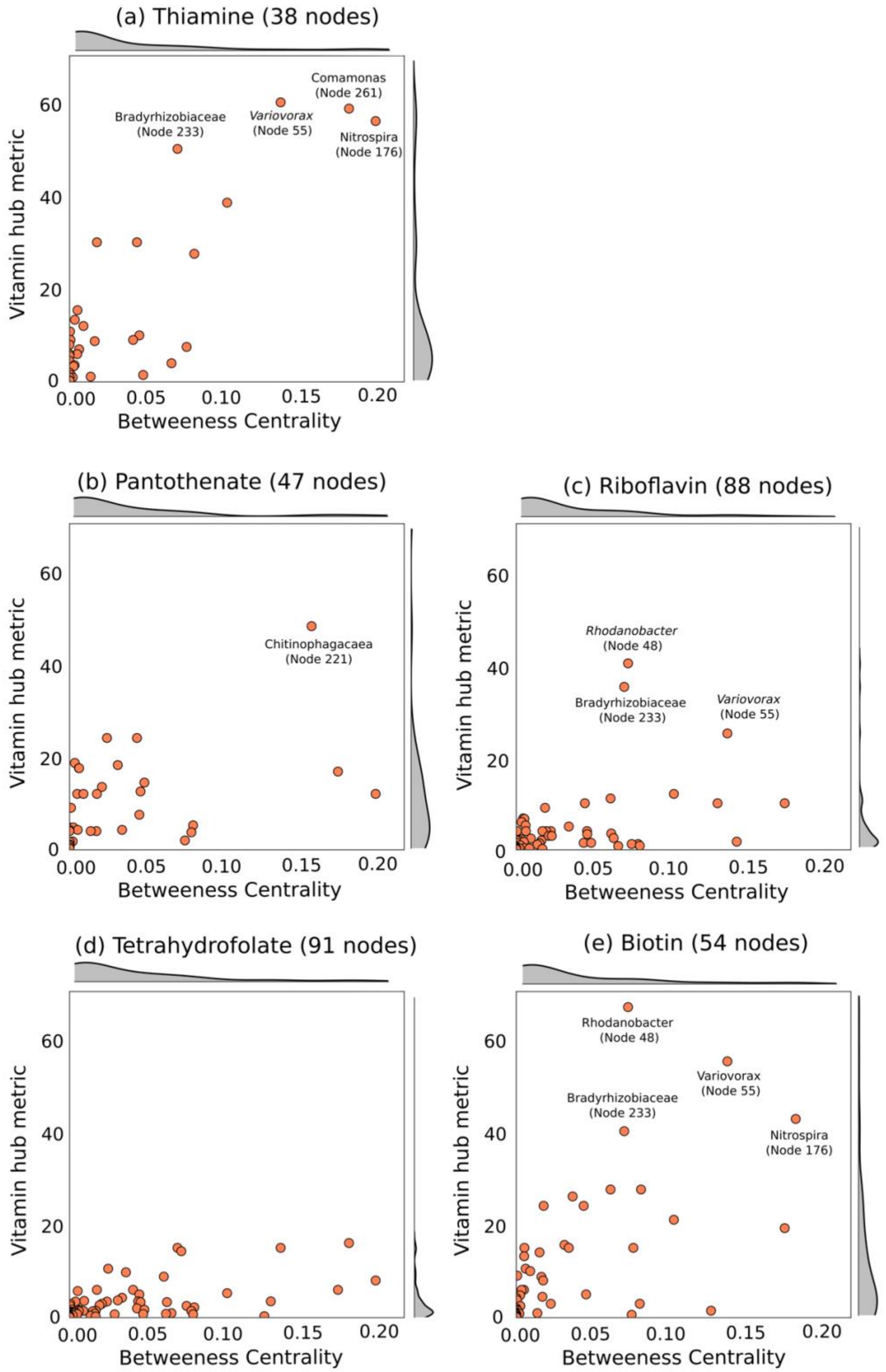

Figure S4. The calculated vitamin hub metric for (a) thiamine, (b) pantothenate, (c) riboflavin, (d) tetrahydrofolate and (e) biotin based on the observed distribution of vitamin producers across the network shown in Figure 2A.

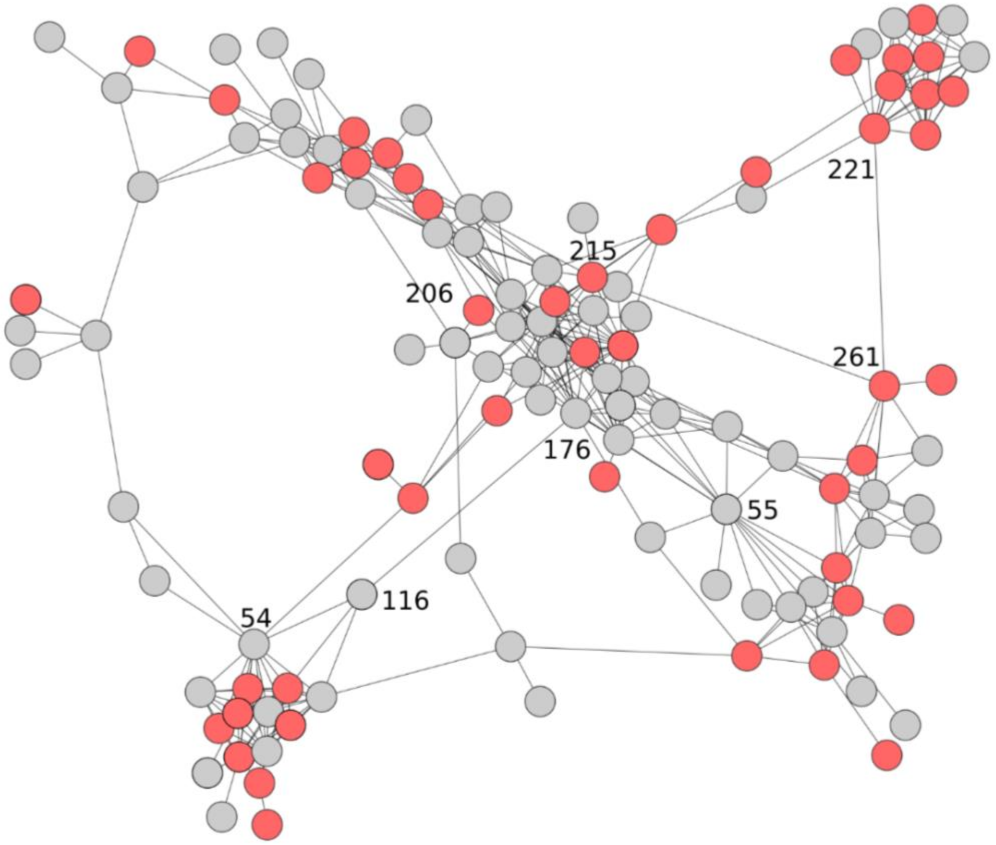

Figure S5. Nodes representing genomes encoding a complete pantothenic acid biosynthetic pathway are shown in red on the correlation network (Figure 2A) derived from the bioreactor metagenomes. Those with an incomplete pathway and, therefore, predicted pantothenic acid auxotrophs, are shown in grey. Nodes determined to be network hubs are labeled with their node numbers.

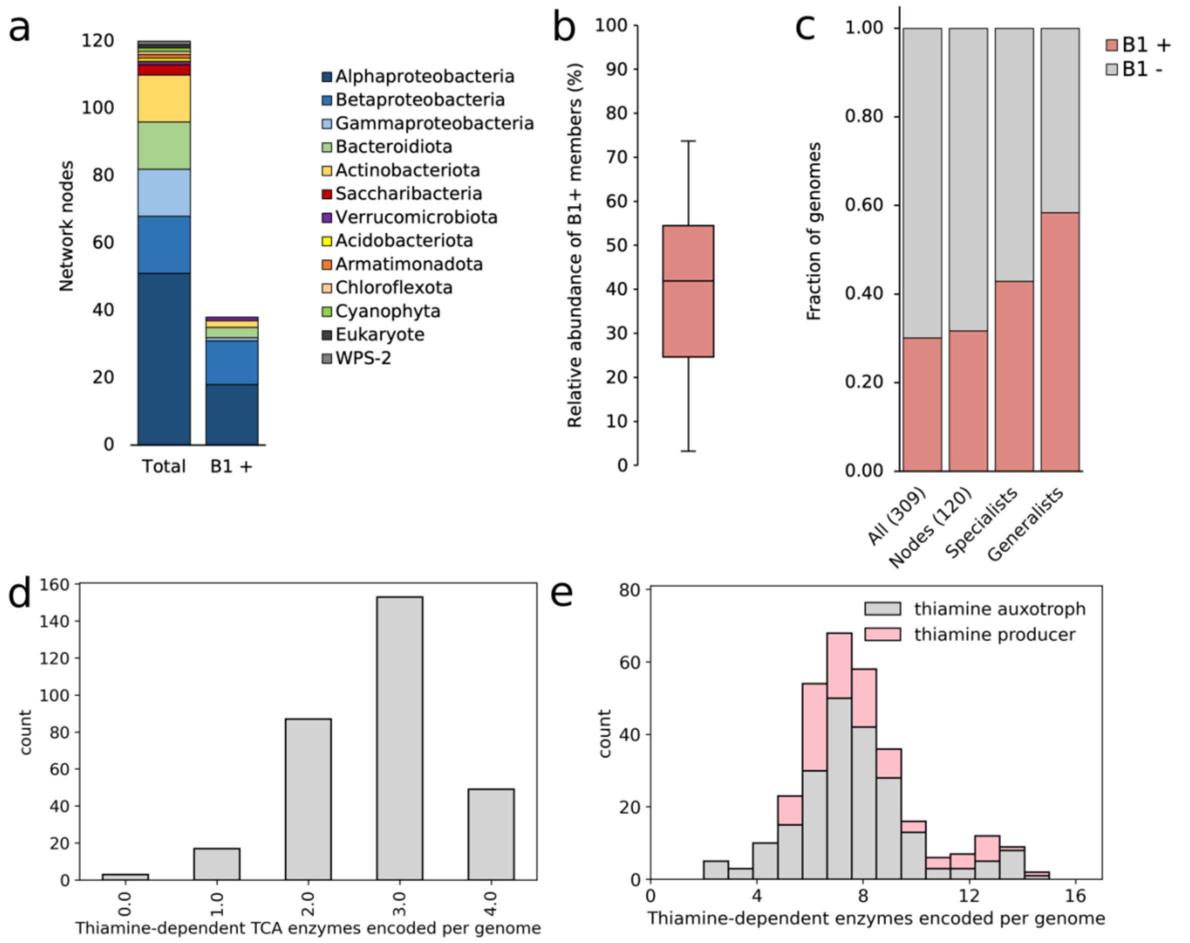

Figure S6. Statistics associated with thiamine producers and consumers from the bioreactor metagenomes. (A) The taxonomy of the organisms represented in the correlation network and those in the network capable of thiamine biosynthesis. (B) The proportion of each of the 92 biologically independent microbial communities that encode for a complete thiamine biosynthesis pathway. Boxplot whiskers represent the minimum and maximum values within 1.5 times the interquartile range, while the bounds of the box indicate the 25th and 75th percentiles, and the center indicating the median. (C) The proportion of all 309 bacteria, the 120 bacteria represented in the network and of the specialists and generalists which endcode their own B1. Generalists are defined as organisms which are found in 75% of the 92 metagenomes and have a mean relative abundance of greater than 1%. Specialists are defined as those which are present in 25% or fewer of the metagenomes but have a mean relative abundance of greater than 1%. (D) The number of thiamine-dependent enzymes, specifically of the TCA, encoded by each genome and the (E) total number of thiamine-dependent enzymes encoded per genome thiamine-auxotroph and thiamine-producing genome. See Supplementary data 4 for a description of these enzymes.

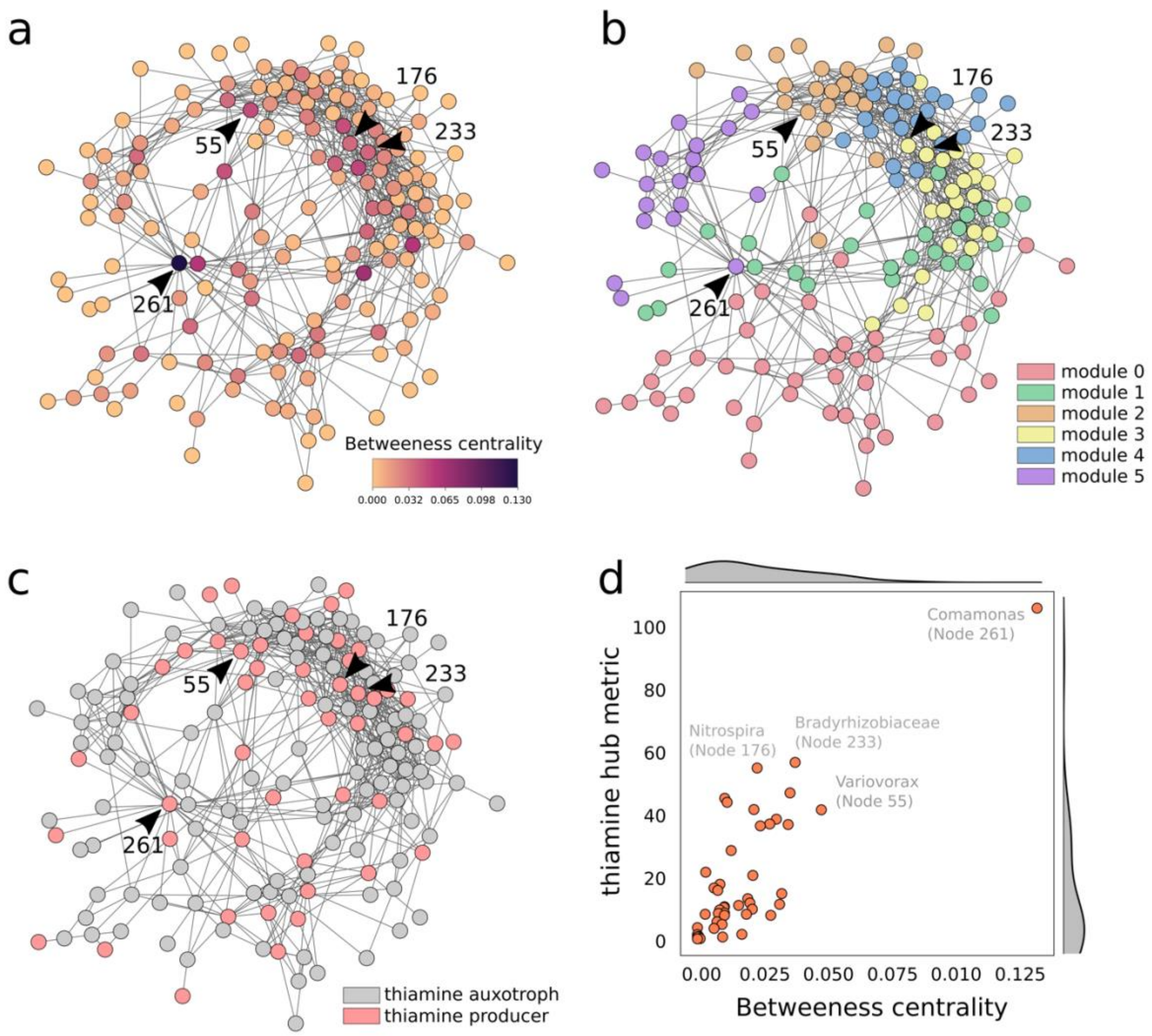

Figure S7. Network analysis performed using correlation determined by Flashweave found *Variovorax* to be a potentiallyimportant node for thiamine provisioning. (A) The betweeness centrality was used to identify network hubs. (B) The network was found to contain six distinct modules. (C) Thiamine biosynthesis was encoded by 49 of the 164 genomes represented in the network. (D) The thiamine hub metric was calculated for each of these 49 nodes and finds the *Comamonas*, Bradyrhizobiaceae, *Nitrospira* and *Variovorax* to be some of the highest scorers in the network.

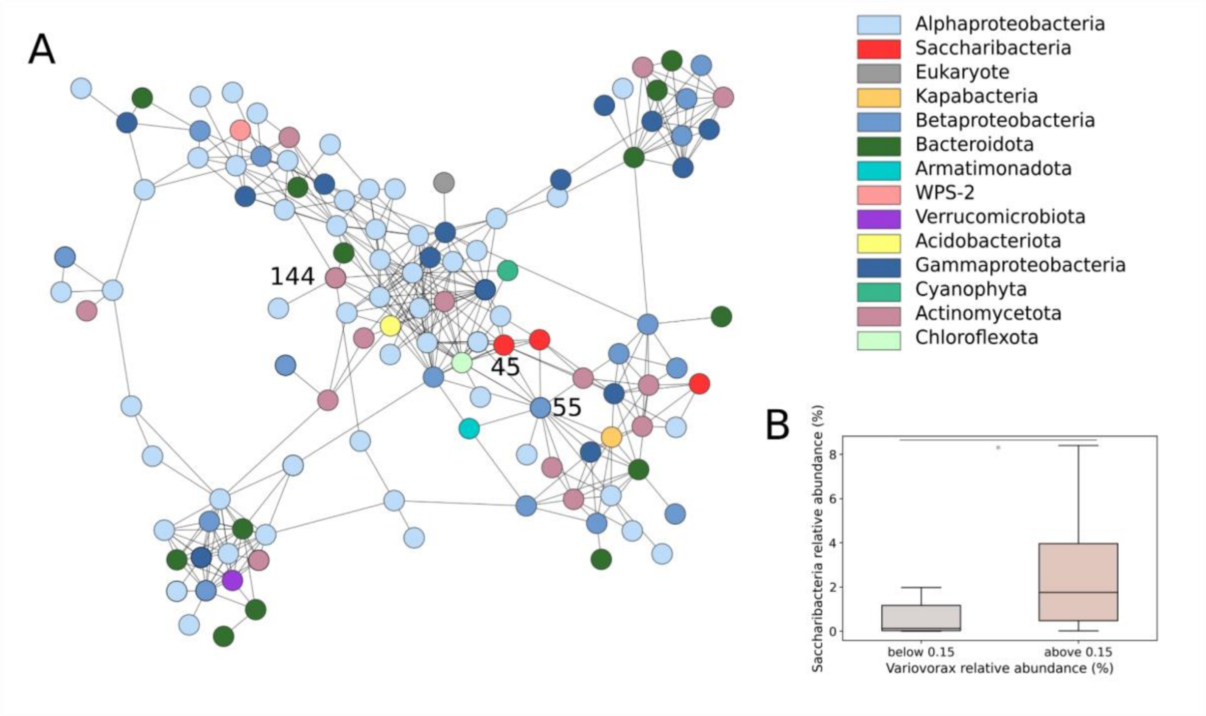

Figure S8. *Variovorax* is predicted to be important for the growth of Saccharibacteria and its *Microbacterium* host. (A) Adapted Figure 4 with greater node separation: *Variovorax* (Node 55) is correlated with a Saccharibacteria (Node 45) as illustrated in the network plot showing phyla annotations. *Microbacterium* (Node 144) and Saccharibacteria both have multiple genes which encode proteins that require thiamine as a cofactor - neither of these genomes encodes for thiamine biosynthesis. (B) The relative abundance of Saccharibacteria (node 45) when *Variovorax* is present in the same sample above and below 0.15 relative abundance (two-tailed independent samples t-test, p=0.015, n=31 biologically independent samples represented by each boxplot). Boxplot whiskers represent the minimum and maximum values within 1.5 times the interquartile range, while the bounds of the box indicate the 25th and 75th percentiles, and the center indicating the median.
