# Supplementary material for: Vitamin interdependencies predicted by metagenomics-informed network analyses and validated in microbial community microcosms
Source: Nat Commun. 2023 Aug 8;14:4768. doi: 10.1038/s41467-023-40360-4 (PMC10409787; doi:10.1038/s41467-023-40360-4)
Supplement: Supplementary file 3 — Description of Additional Supplementary Files [file 41467_2023_40360_MOESM3_ESM.pdf]

Description of Additional Supplementary Files

Supplementary Data 1. Statistics of the modules determined using the Louvian algorithm of the 0.35 cutoff SparCC network.

Supplementary Data 2. Network (SparCC 0.35 correlation cutoff) information relating to the nodes and corresponding MAGs from bioreactor metagenomes.

Supplementary Data 3. Observed growth of *Variovorax* following 48 hour incubation in M9 minimal media in the presence of various B vitamins.

Supplementary Data 4. Enzymes of central metabolism which require thiamine as a cofactor. Adapted from Shelton et al. (22).

Supplementary Data 5. The minimum requirements for the determined presence of a vitamin biosynthetic pathway in a microbial genome.

Supplementary Data 6: Thiaminemonophosphate kinase (ThiL) sequence alignment.
