# Supplementary material for: Vitamin interdependencies predicted by metagenomics-informed network analyses and validated in microbial community microcosms
Source: Nat Commun. 2023 Aug 8;14:4768. doi: 10.1038/s41467-023-40360-4 (PMC10409787; doi:10.1038/s41467-023-40360-4)
Supplement: Supplementary file 6 — Reporting Summary [file 41467_2023_40360_MOESM6_ESM.pdf]

nature portfolio

Corresponding author(s): Jillian F Banfield

Last updated by author(s): Jul 9, 2023

Reporting Summary

Nature Portfolio wishes to improve the reproducibility of the work that we publish. This form provides structure for consistency and transparency in reporting. For further information on Nature Portfolio policies, see our Editorial Policies and the Editorial Policy Checklist.

Statistics

For all statistical analyses, confirm that the following items are present in the figure legend, table legend, main text, or Methods section.

n/a	Confirmed	
☐	☒	The exact sample size (*n*) for each experimental group/condition, given as a discrete number and unit of measurement
☐	☒	A statement on whether measurements were taken from distinct samples or whether the same sample was measured repeatedly
☐	☒	The statistical test(s) used AND whether they are one- or two-sided<br>*Only common tests should be described solely by name; describe more complex techniques in the Methods section.*
☐	☒	A description of all covariates tested
☐	☒	A description of any assumptions or corrections, such as tests of normality and adjustment for multiple comparisons
☐	☒	A full description of the statistical parameters including central tendency (e.g. means) or other basic estimates (e.g. regression coefficient) AND variation (e.g. standard deviation) or associated estimates of uncertainty (e.g. confidence intervals)
☐	☒	For null hypothesis testing, the test statistic (e.g. *F*, *t*, *r*) with confidence intervals, effect sizes, degrees of freedom and *P* value noted<br>*Give P values as exact values whenever suitable.*
☒	☐	For Bayesian analysis, information on the choice of priors and Markov chain Monte Carlo settings
☒	☐	For hierarchical and complex designs, identification of the appropriate level for tests and full reporting of outcomes
☐	☒	Estimates of effect sizes (e.g. Cohen's *d*, Pearson's *r*), indicating how they were calculated

Our web collection on statistics for biologists contains articles on many of the points above.

Software and code

Policy information about availability of computer code

Data collection	No software was used for data collection
Data analysis	dRep v3.0.0 (https://github.com/MrOlm/drep)<br>Hmmscan v3.3 (http://hmmer.org/)<br>CoverM v0.6.1 (https://github.com/wwood/CoverM)<br>FastSpar v1.0.0 (https://github.com/scwatts/fastspar)<br>Flashweave v0.19.2 (https://github.com/meringlab/FlashWeave.jl)<br>GToTree v1.5.31 (https://github.com/AstrobioMike/GToTree)<br>IQtree v1.6.12 (https://github.com/Cibiv/IQ-TREE)<br>iTol v6.5.8 (https://itol.embl.de/)<br>CheckM v1.2.1 (https://github.com/Ecogenomics/CheckM)<br>Sickle (www.github.com/najoshi/sickle)<br>ggKbase (https://ggkbase.berkeley.edu)<br>IDBA-UD v1.1.3 (https://github.com/loneknightpy/idba)

For manuscripts utilizing custom algorithms or software that are central to the research but not yet described in published literature, software must be made available to editors and reviewers. We strongly encourage code deposition in a community repository (e.g. GitHub). See the Nature Portfolio guidelines for submitting code & software for further information.

Data

Policy information about availability of data

All manuscripts must include a data availability statement. This statement should provide the following information, where applicable:

- Accession codes, unique identifiers, or web links for publicly available datasets
- A description of any restrictions on data availability
- For clinical datasets or third party data, please ensure that the statement adheres to our policy

Variovorax and Proteobacterial genomes studied for pathway completeness were recovered from GTDB (r207_v2, https://gtdb.ecogenomic.org/). The 309 dereplicated bioreactor genomes used in this study are available through the online database ggKbase at https://ggkbase.berkeley.edu/SCN_92/organisms. The raw read data and all MAGs from the previous studies and used in this study are available at NCBI (http://www.ncbi.nlm.nih.gov/) under accession codes PRJEB29350 (https://www.ncbi.nlm.nih.gov/bioproject/513540), PRJNA279279 (https://www.ncbi.nlm.nih.gov/bioproject/279279) and PRJNA629336 (https://www.ncbi.nlm.nih.gov/bioproject/?term=PRJNA629336).

Research involving human participants, their data, or biological material

Policy information about studies with human participants or human data. See also policy information about sex, gender (identity/presentation), and sexual orientation and race, ethnicity and racism.

Reporting on sex and gender	n/a
Reporting on race, ethnicity, or other socially relevant groupings	n/a
Population characteristics	n/a
Recruitment	n/a
Ethics oversight	n/a

Note that full information on the approval of the study protocol must also be provided in the manuscript.

Field-specific reporting

Please select the one below that is the best fit for your research. If you are not sure, read the appropriate sections before making your selection.

☒ Life sciences ☐ Behavioural & social sciences ☐ Ecological, evolutionary & environmental sciences

For a reference copy of the document with all sections, see nature.com/documents/nr-reporting-summary-flat.pdf

Life sciences study design

All studies must disclose on these points even when the disclosure is negative.

Sample size	No sample size calculations were performed prior to experimentation. The clonal bacterial and yeast strains used in our study exhibited consistent growth behavior in previous experiments. Based on this reliability and the inclusion of appropriate controls, sample sizes of 4-6 were deemed sufficient.
Data exclusions	No data were excluded.
Replication	All pure- and co-culture microbial culturing experiments were performed with at least 4 replicates. All attempts were successful and are reported. A single metagenomic sampler per microbial community were sampled,with no replicates, in the original studies which are referenced in text.
Randomization	No categorical groupings of bioreactor metagenomes or genomes were performed during network analyses. Bacterial genomes were compared based on the number of a total of six possible vitamin encoded in tier genomes. These categories were compared based on distribution of the lengths of these genomes.
Blinding	No blinding was performed as all measurements were objective and quantifiable with little to no susceptibility of subjective bias.

Reporting for specific materials, systems and methods

We require information from authors about some types of materials, experimental systems and methods used in many studies. Here, indicate whether each material, system or method listed is relevant to your study. If you are not sure if a list item applies to your research, read the appropriate section before selecting a response.

Materials & experimental systems

n/a	Involved in the study
☒	☐ Antibodies
☒	☐ Eukaryotic cell lines
☒	☐ Palaeontology and archaeology
☒	☐ Animals and other organisms
☒	☐ Clinical data
☒	☐ Dual use research of concern
☒	☐ Plants

Methods

n/a	Involved in the study
☒	☐ ChIP-seq
☒	☐ Flow cytometry
☒	☐ MRI-based neuroimaging
